# Factors associated with suicide attempt risk in adolescent inpatient psychiatric care: toward a practical model

**DOI:** 10.1007/s00787-023-02272-y

**Published:** 2023-09-05

**Authors:** Rafał Szmajda, Łukasz Mokros, Dagmara Szmajda-Krygier, Agnieszka Gmitrowicz

**Affiliations:** 1 Medical University of Lodz, Łódź, Poland; 2 Institute of Psychiatry and Neurology, Warsaw, Poland

**Keywords:** Suicide attempt, Adolescents, Inpatients, Mental disorders

## Abstract

Suicide is an important social and medical problem, particularly among children and adolescents. The aim of the study was to determine the association of the psychiatric diagnosis and selected psychosocial factors with the risk of suicide attempts among patients of an adolescent psychiatric unit. A retrospective analysis was performed on a database of consecutive *N* = 1311 patients aged 13–18 years of the adolescent psychiatric ward. A hierarchical logistic regression analysis was performed to assess the predictive value of the main psychiatric diagnosis, for factors selected from the database to determine their influence on the relative risk of a suicide attempt. Primary diagnoses of mood disorders and emotional and behavioral disorders were associated with an increased risk of a current admission after a suicidal attempt, a history of past suicidal attempts and non-suicidal self-harm (NSSI). History of NSSI was associated with a fourfold increase probability of a suicide attempt. Truancy, sexual abuse, heartbreak and frequent conflicts were related to a rise in suicidal attempt risk. Learning difficulties were found to be linked to increased probability of suicidal attempt, but only among women. The current study confirms that the primary diagnosis, NSSI and well-recognized psychosocial factors (including family- and school-related factors) may prove useful in the assessment of suicidal risk among adolescents admitted to a psychiatric ward.

## Introduction

Suicide is a social and medical problem that continues to gain meaning. According to data from the World Health Organization (WHO), the average suicide rate is 10.6 per 100,000, which means that on average every 40 s, one person in the world dies by suicide [1].

Suicide is not only about the tragedy of an individual, because in its own multidimensionality it also affects its relatives, family, partners, friends and acquaintances [2, 3, 24]. Moreover it also has emotional impact on medical personnel involved in helping a person in a suicidal crisis [4].

With regard to medical practice, psychiatrists and psychiatrists of children and adolescents most often encounter the problem of suicide, and the responsibility is of these specialists to carefully assess the risk of suicide, to work with patients in a suicide crisis and to make consultation recommendations for other specialties in the states of increased risk of suicide.

For practicing physicians in the assessment of a patient in crisis, it is important to know the risk factors, including the suicide risk of patients with a mental disorder [5, 24].

As research on suicidal ideation shows that they might have been experienced by every three teenagers, and 6–9% of them confirmed that they had completed a suicide attempt [6].

Over the course of 20 years, the psychiatry of children and adolescents has changed. One can observe different distributions of diagnoses in the patient population. Due to the increase in the number of suicide attempts and deliberate self-harm in adolescents, more numbers are now admitted to hospitals. This forces the search for effective evaluation methods of suicide risk in the adolescent population [5].

The aim of the study was to determine the association of the psychiatric diagnosis and selected psychosocial factors with the risk of suicide attempts among patients of an adolescent psychiatric unit.

## Methods

### Study sample and design

The material for the research was a retrospective database of patients aged 13–18 years, made as part of institutional grant activities of the Department of Youth Psychiatry of the Central Teaching Hospital, Medical University of Lodz, Poland. The database included consecutive patients of the adolescent psychiatry unit in the years 2006–2015. Total sample selected from the database included 1311 items corresponding to such a number of patients. Patient data were assembled according to the categories of diagnosis groups based on the WHO International Classification of Diseases, 10th edition (ICD-10). The study sample was divided into two groups:

- Study group—patients with a suicide attempt on admission (with an additional distinction based on the number of past suicide attempts).
- Control group—patients with no history of suicide attempt on admission.

The utilized database comprised clinical and psychosocial factors, which were hypothesized to be linked to the risk of suicide attempt. The selected factors included: family burden (in the form of schizophrenia, bipolar disorder, recurrent depressive disorders, family suicide attempts, use of psychoactive substances in the family), living conditions (with a parent, caregiver, in an institution, e.g., at home children, others, youth centers of sociotherapy or education), experience of stressful life events (physical abuse, sexual harassment, loss of a parent before the age of 15, rejection by peers, and heartbreak), family difficulties (divorce/separation, crime in the family, rivalry with siblings, frequent conflicts, lack of support in the family), and school difficulties (truancy, repeating grades, learning difficulties, dropping out of school). A group of patients with non-suicidal self-injuries was also selected, and then self-mutilation was included in the statistical models as a separate diagnosis according to the category in ICD-10 as X78—intentional self-harm by a sharp object.

### Statistical analysis

A number of logistic regression models were developed, taking into account the typed risk factors individually and together in their influence on the probability of a suicide attempt. The influence of the age of the onset of mental disorders on the risk of suicide attempt was also assessed. The risk was expressed as odds ratios (ORs) and 95% confidence intervals (CIs).

The analyses were performed in IBM SPSS Statistics, version 25. Categorical data were characterized with numbers (*n*) and percentages (%), and continuous data with means (*M*) and standard deviations (SD). The Chi-square test was performed to assess contingencies in 2 × 2 tables. Hierarchical logistic regression models were constructed for prediction of suicide attempt. Also, a stepwise approach was assumed to elucidate the effects of single factors in the greatest precision. The categorical variables were included in the models by means of simple coding, with the first category being the referential one. The goodness of fit of the logistic regression models was assessed with Nagelkerke *R*^2^, *χ*^2^ test for a whole model. The assumed level of significance was alpha = 0.05.

### Ethical considerations

This study was conducted in accordance with the Declaration of Helsinki. The bioethics committee did not express its objection to the use of this anonymous database.

## Results

### Group characteristics

The group was mostly female (60.8%, *p* < 0.001). The most common diagnoses concerned the groups of neurotic, stress-related and somatoform disorders (F40–49) and emotional and behavioral disorders (F90–98) of the ICD-10. Suicide attempts were found in 487 examined patients (37.2%), while NSSI concerned 572 patients (43.7%). Every third respondent indicated a family burden of substance abuse in the nuclear family.

The mean duration of hospitalization was 35 days. The longest stay in the study population was 265 days. On average, patients were hospitalized twice, while the highest number of hospitalizations was 13. The mean age of onset of mental disorders was 14 years. The detailed characteristics of the study group is in Table 1.

**Table 1:** The clinical and psychosocial characteristics of the studied group of patients of the adolescent psychiatric unit (*N* = 1311)

Age of onset of mental disorders, M ± SD	13.6 ± 3.1
Duration of hospitalization (days), M ± SD	34.9 ± 127.6
Number of hospitalizations, M ± SD	2 ± 1
*Main diagnosis, N (%)*	
F00–09. Organic mental disorders	32 (2%)
F10–19. Substance use disorders	51 (4%)
F20–29. Schizophrenia and related disorders	206 (16%)
F30–39. Mood disorders	177 (13%)
F40–49. Neurotic, stress-related and somatoform	379 (29%)
F50. Eating disorders	48 (4%)
F60–61. Personality disorders	6 (1%)
F70–79. Intellectual disability	76 (6%)
F80–89. Disorders of psychological development	24 (2%)
F90–98. Behavioral and emotional disorders	280 (21%)
Other	32 (2%)
History of suicide attempts, *N* (%)	487 (37%)
History of NSSI, *N* (%)	572 (44%)
*Family burden, N(%)*	
None	676 (52%)
Schizophrenia	84 (6%)
Bipolar disorder	23 (2%)
Recurrent depressive disorder	68 (5%)
Suicide	51 (4%)
Substance abuse	407 (31%)
*Living conditions, N (%)*	
With parents	1047 (80%)
With caretaker	82 (6%)
Institutionally reared	136 (105)
Other	44 (3%)

*M* mean, *SD* standard deviation, *N* number of observations, *NSSI* non-suicidal self-injury

The probability of a current admission after a suicidal attempt was increased 1.7-fold for mood disorders and 2-fold for behavioral and emotional disorders, and decreased 2.4-fold for schizophrenia and related disorders. F30–39, F40–48, and F90–98 diagnostic categories were associated with an increase in the risk of a history of past suicide attempts, while F20–29, F50, F70–79, and F80–89 were linked to a fall in the probability of a history of past suicide attempts. The risk of a history of non-suicidal self-injury was associated with the diagnoses of F00–09, F20–29, F30–39, and F90–98 (Table 2).

**Table 2:** Results of univariate logistic regression models for each group of diagnoses according to the International Classification of Diseases, 10th edition (ICD-10) as a risk factor for admission to a psychiatric hospital after a suicide attempt, past history of suicide attempts, and history of non-suicidal self-injury in the studied group of patients of an adolescent psychiatric ward

Primary diagnosis	Current admission after a suicide attempt	History of past suicide attempts	History of non-suicidal self-injury
F00–09	0.41 (0.18–0.93)	0.47 (0.20–1.08)	0.42* (0.19–0.95)
F10–19	0.88 (0.50–1.56)	0.77 (0.42–1.40)	0.83 (0.47–1.47)
F20–29	0.42* (0.30–0.58)	0.41* (0.28–0.57)	0.43* (0.31–0.59)
F30–39	1.65* (1.19–2.26)	1.89* (1.37–2.60)	1.64* (1.19–2.25)
F40–48	0.97 (0.75–1.24)	1.50* (1.18–1.92)	0.92 (0.72–1.17)
F50	0.68 (0.38–1.25)	0.43* (0.21–0.88)	0.77 (0.42–1.39)
F60–61	0.82 (0.51–1.31)	0.84 (0.15–4.63)	1.29 (0.26–6.42)
F70–79	0.82 (0.51–1.31)	0.24* (0.12–0.47)	0.83 (0.52–1.34)
F80–89	0.63 (0.27–1.48)	0.24* (0.07–0.80)	0.64 (0.27–1.51)
F90–98	1.99* (1.52–2.59)	1.57* (1.20–2.06)	2.00* (1.53–2.61)
Other	0.86 (0.42–1.77)	0.88 (0.42–1.85)	0.93 (0.45–1.92)

Presented as odds ratios with 95% confidence intervals

The ICD-10 diagnoses group abbreviations are explained in Table 1. *p < 0.05

*p* test probability

Most of the suicide attempts in the study group were made by cutting the skin. The second most frequent method chosen was the intentional use of drugs. The least frequently chosen method was choking (understood as a strangulation, an attempt to hang).

There were statistically significant associations between the selected psychosocial variables and the probability of a suicide attempt. The greatest influence on making a suicide attempt was demonstrated for heartbreak (2.2-fold) (Model 1 in Table 3).

**Table 3:** The results of consecutive, stepwise multivariate logistic regression models predicting the probability of attempting suicide, taking into account chosen clinically significant psychosocial stressors, primary diagnoses, self-harm, female sex and found interactions as risk factors in the studied group of patients of the adolescent psychiatric ward

Variable	Model 1	Model 2	Model 3	Model 4	Model 5	Model 6	Model 7
Truancy	1.96†	2.22†	2.05†	2.43†	1.84†	1.81†	1.86†
Learning difficulties	0.61†	0.65**	0.64**	0.65**	0.63†	1.70*	1.74*
Sexual abuse	2.16†	1.79**	1.76*	1.87**	1.73*	1.64†	1.65†
Heartbreak	2.19†	1.94**	1.94†	1.95†	1.64†	–	–
Rejection by peers	0.71**	0.77*	0.77*	–	–	–	–
Frequent conflicts	1.52**	1.39*	1.39**	–	–	–	–
Female sex	–	2.47**	2.56**	2.47†	1.86†	–	–
Primary diagnosis F90–98	–	–	1.39*	–	–	–	–
Primary diagnosis F30–39	–	–	–	1.92†	–	–	1.68**
Non-suicidal self-harm	–	–	–	–	4.24†	4.34†	4.24†
Sex * learning difficulties						2.04*	1.98*

Presented as odds ratios with marked probability in the test

*p < 0.05, **p < 0.01, †*p* < 0.001

When taking sex into account, the individual predictors remained statistically significant. Female sex was associated with a 2.5-fold increase in risk of suicide attempt. In other words, gender has an influence on the risk of attempting suicide regardless of the factors related to family and school circumstances (Model 2 in Table 3).

Another model included the same factors of the family situation, gender and the F90–98 diagnoses. Female sex still had the greatest influence on the risk of attempting suicide (Model 3 in Table 3)).

However, the F30–39 group was associated with an increased risk of suicide attempt and was the third predictor after female sex and truancy. Here, only peer rejection and frequent conflicts ceased to be a statistically significant predictor (Model 4 in Table 3).

Age was not a statistically significant predictor of suicide attempts (hence it was not analyzed further).

The model with a lesser influence on the risk of suicide attempt is the self-harm model. People who had self-injuries in the presence of other statistically significant predictors had a fourfold greater chance of attempting suicide (Model 5 in Table 3).

A statistically significant interaction of sex of the respondents concerned only learning difficulties. The influence of learning difficulties on the risk of suicide attempt concerned mainly women. The factor "sex * learning difficulties " was more strongly associated with the risk of attempting suicide than sex alone or learning difficulties (Model 6 in Table 3).

There were no other statistically significant interactions between the sex of the subjects and self-harm with factors related to the family situation. The only statistically significant interaction influencing the attempted suicide was the sex interaction of the respondents with learning difficulties.

In the final model, self-harm had the greatest impact on suicidal risk, followed by learning difficulties in women, and truancy in the third place (Model 7 in Table 3).

## Discussion

The presented research results show that the risk of attempting suicide and self-harm is different for the examined groups of psychiatric diagnoses.

It should be noted that this work is of an observational and retrospective nature. This means that it is only possible to determine the relationship between the given factors and the probability of attempting a suicide, and not the influence of these factors on the risk of a suicide attempt. The description of the results uses the formulation of the influence of given factors on the risk; however, it is only about the impact in the statistical sense, resulting from the choice of the method (logistic regression) and the construction of the models.

In the studied population, suicide and self-harm attempts most often concerned people diagnosed with mood disorders (groups F30 to F39). Half of those hospitalized at any given time with these diagnoses had at least one history of suicide attempt. In the studied population, suicide attempts were most often made by patients diagnosed with affective disorders (50.6% of the respondents had a history of a suicide attempt). In studies of the general population diagnosed with affective disorders, this percentage was 66 [7–9]. The difference in the percentage for both these populations should be noted. Despite the high risk of suicide attempts in the population of children and adolescents with affective disorders, the calculated risk is lower than in studies on the general population.

The second group with which suicide attempt was most often associated was the group of diagnoses with neurotic, stress-related and somatic disorders. It should be noted, however, that in the studied population, one of the most common diagnoses are adjustment disorders, defined by the ICD code F43.2, which in their picture often run as a depressive reaction (short-term or prolonged). Considering the additionally objectively higher share of reactive factors in adolescent depression, it should be assumed that the group of affective disorders and neurotic disorders in the study may partially permeate one another.

In the study, the estimated risk of a suicide attempt in patients from the group of psychotic disorders was 44%, which is comparable with the data from the general population, where the risk of a suicide attempt related to the occurrence of schizophrenia ranges from 20 to 45% [10].

The risk of a suicide attempt in addiction is similar for the general population and the studied population and amounts to 16% (the literature reports 7–15% for the general population) [11].

In the group of respondents with intellectual disability, the risk of a suicide attempt was lower than that in the adult population. In the cited studies, the risk of suicide attempts for the F70–F79 population was 19% [12]. The results of this study show that 13% of patients attempted suicide.

The undertaken study showed a high risk of suicide attempts for the population of patients diagnosed with "Behavior and emotional disturbances usually starting in childhood and adolescence". It is a group of diagnoses specific for the population of children and adolescents, which includes, among others, ADHD and behavioral disorders. The high percentage of suicide attempts in this subpopulation may indicate a suicide risk associated with the repeatedly described impaired impulse control in patients associated with these mental disorders [13].

In the eating disorders group (F50–F59), the risk of a suicide attempt was 20% and differs from that calculated for the adult population—amounting to 26% [14, 15].

The diagnosis groups F80–F89 and F60–F61 contained three and two probants, respectively, which did not allow to draw reliable conclusions on the basis of such a small group.

Based on the results, the hypothesis is confirmed that the risk of a suicide attempt depends on the basic diagnosis in children and adolescents.

Self-injuring persons showed a four times higher risk of suicide in the presence of other unfavorable predictors. Moreover, in subjects with a history of NSSI, the method of suicide attempts by cutting was significantly more frequent. On the basis of the created statistical model taking into account the predictive factors to the greatest extent for undertaking a suicide attempt, the presence of self-harm in the first place (as the most important factor) was demonstrated.

Among the school difficulties, only truancy turned out to be a statistically significant risk factor for suicide attempt.

Among the examined family factors affecting the risk of suicide attempts, two were statistically significant: frequent conflicts in the family and lack of support in the family.

The heartbreak turned out to be a statistically significant risk factor for the minor's attempted suicide.

The type of suicide attempt also depends on the diagnosis made. As shown, in the group of people who overdose on drugs, people diagnosed with F40–48 dominate, while people diagnosed with cuts—F90–98. Moreover, statistically significant dependencies of the type of suicide attempt concern the following parameters of family situation: truancy, dropout, lack of support in the family. People who played truant attempted suicide mainly through the use of drugs and suffocation. The same goes for people leaving school. As for people without family support, they mainly strangled and cut. Other trial types of predictors were: people who were victims of physical violence mostly attempted suicide by jumping from a height or under a vehicle and taking medication. The same is true for people with a heartbreak. According to the obtained results, the most common method of suicide attempt was cutting oneself, while the second most frequent method was taking medications.

The majority of respondents who attempted suicide were women, regardless of the type of suicide attempt.

Additionally, the performed logistic regression models showed other dependencies. For the logistic regression modeling of individual diagnoses according to ICD groups, in relation to the entire studied population, the odds ratio is the highest for groups F80–F89 and F30–F39. It is difficult to interpret the odds ratio for groups F80–F89, as this group is heterogeneous in the ICD-10 Classification. It includes both pervasive developmental disorders (i.e., the autism spectrum) and partial deficits, e.g., developmental dyslexia. At the same time, it should be noted that developmental dyslexia, as an isolated deficit, will rarely cause psychiatric hospitalization, and the study group, despite adopting the separation criteria, consisted mainly of patients with a diagnosis of autism spectrum disorder. A significant part of the studies assessing suicide risk was carried out on small groups, so it is difficult to draw significant statistical conclusions on this basis. Significant data were provided by a 2013 study by Mayes et al. conducted on a population of 791 children on autism spectrum. The study compared children with autism with a group of children with depressive symptoms and a group of children without mental disorders. The study showed a 28-fold higher risk of suicidal behavior in the group of patients with pervasive developmental disorders [16]. The present study also shows the highest risk of suicide attempt in patients from groups F80 to F89. The odds ratio for this group was 1.98 and it was the highest among all models.

The second group with the highest odds ratio is the group of mood disorders—numerous studies have shown that mood disorders are a group with very high values of suicide risk [1, 17]. In this study, the odds ratio in the group of mood disorders was 1.64. The quotient was calculated in relation to the control group consisting of other patients. Despite the specific group in which the risk is still higher than in the general population, mood disorders, both unipolar and bipolar, still constitute a group with a higher than control risk value.

The results described above show how high the risk of suicide attempts is in the population of autism spectrum disorder and mood disorders. While drawing conclusions on the basis of the heterogeneous groups F80–F89 in this study is debatable, the very high risk in the group of mood disorders is confirmed in all studies conducted so far.

Then, statistical models were created taking into account the risk of a suicide attempt for various predictors from the analyzed database. As the analysis shows, the highest odds ratio for a suicide attempt is characterized by the stressor of an emotional profession, while sexual abuse is the second with the highest risk.

To the author's knowledge, only a few works analyzing the suicide risk of adolescents in the face of a heartbreak have been published so far. This paper shows how important this factor is, and that clinicians and practitioners should pay more attention to the crisis in the romantic relationship as an important risk factor for suicidal behavior. There are studies showing an increased risk of suicidal behavior related to heartbreak, but they usually concern the population over 18 years of age [18, 19]. In 2013, Karch, in her analysis of suicides among adolescents in the USA, indicated heartbreak as the third most important factor in a completed suicide [20]. Another perspective was shown by an Iranian research, where adolescents aged 14–17 years declared heartbreak as the main reason for making the attempt [21].

Sexual abuse is the second most significant factor in the odds ratio. Numerous studies indicate a relationship between experienced sexual trauma and an increased chance of suicidal behavior [22–24].

When modeling in which another variable, sex, was assumed, the significant predictors of suicide risk did not change, only the value of the odds ratio changed. However, with the presence of other factors, it is the female sex that shows the highest odds ratio for a suicide attempt.

People who self-injure in the presence of other statistically significant predictors have a 4.24 times greater chance of attempting a suicide. This again demonstrates self-harm as an independent risk factor for suicide attempts.

Among the factors of the family and school situation, the statistically significant gender interaction of the respondents concerns only learning difficulties. The influence of learning difficulties on the risk of committing a suicide attempt concerns mainly women. It is noteworthy that the interaction of gender with learning difficulties is the second factor after self-harm, i.e., it influences the risk of attempting suicide.

The current study confirms that the primary diagnosis, NSSI and well-recognized psychosocial factors (including family- and school-related factors) may prove useful in the assessment of suicidal risk among adolescents admitted to a psychiatric ward.

The obtained results, together with the created statistical model of factors with mutual influence and the greatest importance for the risk of suicide attempt, constitute an important juxtaposition that may characterize the group with the highest degree of suicide risk and may allow for the identification of the patient most at risk of suicidal behavior at the emergency room level. In the future, this may allow the creation of a dispersing group with the highest risk, which should be given special attention.

## Data Availability

Raw data is available upon reasoneable request from the corresponding author.
